# Behavioral Plasticity of Audiovisual Perception: Rapid Recalibration of Temporal Sensitivity but Not Perceptual Binding Following Adult-Onset Hearing Loss

**DOI:** 10.3389/fnbeh.2018.00256

**Published:** 2018-10-31

**Authors:** Ashley L. Schormans, Brian L. Allman

**Affiliations:** Department of Anatomy and Cell Biology, Schulich School of Medicine and Dentistry, University of Western Ontario, London, ON, Canada

**Keywords:** audiovisual perception, temporal order judgment, synchrony judgment, temporal recalibration, multisensory processing, hearing loss, noise exposure, rat

## Abstract

The ability to accurately integrate or bind stimuli from more than one sensory modality is highly dependent on the features of the stimuli, such as their intensity and relative timing. Previous studies have demonstrated that the ability to perceptually bind stimuli is impaired in various clinical conditions such as autism, dyslexia, schizophrenia, as well as aging. However, it remains unknown if adult-onset hearing loss, separate from aging, influences audiovisual temporal acuity. In the present study, rats were trained using appetitive operant conditioning to perform an audiovisual temporal order judgment (TOJ) task or synchrony judgment (SJ) task in order to investigate the nature and extent that audiovisual temporal acuity is affected by adult-onset hearing loss, with a specific focus on the time-course of perceptual changes following loud noise exposure. In our first series of experiments, we found that audiovisual temporal acuity in normal-hearing rats was influenced by sound intensity, such that when a quieter sound was presented, the rats were biased to perceive the audiovisual stimuli as asynchronous (SJ task), or as though the visual stimulus was presented first (TOJ task). Psychophysical testing demonstrated that noise-induced hearing loss did not alter the rats’ temporal sensitivity 2–3 weeks post-noise exposure, despite rats showing an initial difficulty in differentiating the temporal order of audiovisual stimuli. Furthermore, consistent with normal-hearing rats, the timing at which the stimuli were perceived as simultaneous (i.e., the point of subjective simultaneity, PSS) remained sensitive to sound intensity following hearing loss. Contrary to the TOJ task, hearing loss resulted in persistent impairments in asynchrony detection during the SJ task, such that a greater proportion of trials were now perceived as synchronous. Moreover, psychophysical testing found that noise-exposed rats had altered audiovisual synchrony perception, consistent with impaired audiovisual perceptual binding (e.g., an increase in the temporal window of integration on the right side of simultaneity; right temporal binding window (TBW)). Ultimately, our collective results show for the first time that adult-onset hearing loss leads to behavioral plasticity of audiovisual perception, characterized by a rapid recalibration of temporal sensitivity but a persistent impairment in the perceptual binding of audiovisual stimuli.

## Introduction

In order to create an unified percept of objects or events within our external environment, our brain must be able to accurately integrate or bind stimuli from more than one sensory modality (e.g., hearing and vision). Decades of research in numerous species has confirmed that the successful integration of multisensory information is highly dependent upon the features of the unimodal stimuli presented, most notably their intensity and spatiotemporal alignment (King and Palmer, 1985; Meredith and Stein, 1986, 1996; Meredith et al., 1987; Stein and Meredith, 1993; Perrault et al., 2005; Stanford et al., 2005; Rowland and Stein, 2008; Miller et al., 2015). For example, in such cases when an auditory and visual stimulus occur within ~100 ms of each other, the stimuli can be perceived by the observer as having occurred at the same moment in time even though the stimuli were physically asynchronous. Although this integration of closely-timed audiovisual stimuli can offer certain behavioral advantages, such as improved detection, identification and localization of objects in the environment (Hershenson, 1962; Diederich and Colonius, 2004; Hirokawa et al., 2008; Gleiss and Kayser, 2012; Raposo et al., 2012; Siemann et al., 2015), an overly broad window of temporal integration could be problematic, as information from truly separate events may not be correctly perceived as such (Basharat et al., 2018).

The ability to judge the timing of audiovisual stimuli has been well studied in humans using psychophysical testing (for review see Spence et al., 2001; Navarra et al., 2005; Stekelenburg and Vroomen, 2007; Vatakis and Spence, 2007; van Eijk et al., 2008; Vroomen and Keetels, 2010; Keetels and Vroomen, 2012; Stevenson and Wallace, 2013), and more recently in rats trained with appetitive operant conditioning (Schormans et al., 2017a). The two most widely used paradigms to assess audiovisual temporal acuity involve presenting the stimuli at varying stimulus onset asynchronies (SOAs), and requiring participants to judge which modality was presented first (i.e., temporal order judgment, TOJ), or whether the stimuli were presented at the same time or not (i.e., synchrony judgment, SJ). In addition to measuring overall performance during TOJ tasks, researchers often determine the actual timing of the audiovisual stimuli when the participant was most unsure of the temporal order (i.e., point of subjective simultaneity, PSS), as well as the smallest timing interval that could be detected reliably (i.e., just noticeable difference, JND; Vatakis et al., 2008a; Vroomen and Stekelenburg, 2011; Keetels and Vroomen, 2012). In an SJ task such as the flash-beep paradigm, when participants are asked to judge whether or not the visual and auditory stimuli were presented synchronously or asynchronously, researchers can calculate the participant’s temporal binding window (TBW); the epoch of time over which physically asynchronous stimuli are perceived as synchronous (for review see Wallace and Stevenson, 2014). Thus, the TBW provides insight into the degree of temporal tolerance in which asynchronous audiovisual stimuli are likely to be integrated and perceptually bound (Krueger Fister et al., 2016).

Audiovisual temporal acuity normally undergoes fine-tuning throughout childhood and adolescence (Hillock et al., 2011; Hillock-Dunn and Wallace, 2012; Lewkowicz and Flom, 2014; Kaganovich, 2016), making this perceptual ability susceptible to disruption in individuals with developmental disabilities, such as autism spectrum disorder (Bebko et al., 2006; Foss-Feig et al., 2010; Kwakye et al., 2011; de Boer-Schellekens et al., 2013; Stevenson et al., 2014a,b), dyslexia (Hairston et al., 2005; Wallace and Stevenson, 2014) and schizophrenia (Foucher et al., 2007; Carroll et al., 2008; Martin et al., 2013; Stekelenburg et al., 2013; Haß et al., 2017; Stevenson et al., 2017). In such cases, atypical audiovisual temporal acuity often manifests as an increased length of time over which audiovisual stimuli are perceptually bound (i.e., the TBW is wider). Later in life, the ability to accurately perceive the timing of audiovisual stimuli can also be affected, whereby older participants typically show impairments in their perception of temporal order as well as their ability to judge simultaneity (Setti et al., 2011; Chan et al., 2014a,b; Bedard and Barnett-Cowan, 2016; Basharat et al., 2018). Overall, it is clear that the ability to integrate and perceptually bind audiovisual stimuli can vary widely across individuals, as well as shift throughout one’s lifespan. What remains unknown, however, is how adult-onset hearing loss, separate from aging, affects audiovisual temporal acuity. This is an important topic given the prevalence of hearing impairment in younger individuals, often caused by excessive exposure to loud noise at work or during recreational activities. For example, ~12% of children and young adults in the U.S. suffer from noise-induced hearing threshold shifts (Lin et al., 2011), and it is estimated that 22 million U.S. workers are exposed to hazardous noise each year (Tak et al., 2009).

It would be reasonable to predict that moderate hearing loss—which reduces one’s sensitivity to environmental sounds—could distort audiovisual temporal acuity due to the fact that varying the intensity (effectiveness) of auditory and/or visual stimuli is known to alter perceptual judgments in normal-hearing participants (Smith, 1933; Neumann et al., 1992; Neumann and Niepel, 2004; Boenke et al., 2009; Krueger Fister et al., 2016). That said, it is well-established that the perceptual binding of audiovisual stimuli is highly-adaptive to experience, as evidenced from research on participants who were passively exposed to asynchronous audiovisual stimuli (Fujisaki et al., 2004; Navarra et al., 2005; Vatakis et al., 2007, 2008b), as well as those actively engaged in perceptual training (Powers et al., 2009; De Niear et al., 2016, 2018). Thus, an alternative prediction could be that individuals who experience adult-onset hearing loss may show limited changes to their audiovisual temporal acuity, owed to a recalibration of their perceptual ability as they adapt to their permanent hearing impairment.

In the present study, we used a rat model to investigate, for the first time, the nature and extent that audiovisual temporal acuity is affected by adult-onset hearing loss, with specific focus on the time-course of perceptual changes following loud noise exposure. Using appetitive operant conditioning, separate groups of rats were trained to either determine the temporal order of audiovisual stimuli (TOJ task), or differentiate whether audiovisual stimuli were presented synchronously or not (SJ task). In the first experimental series, psychophysical testing was completed for both behavioral tasks in which the intensity of the auditory stimulus was modulated, while the intensity of the visual stimulus was held constant. In the second experimental series, rats trained on the TOJ and SJ tasks were exposed to a loud noise known to cause permanent hearing loss (Schormans et al., 2017b, 2018), and their behavioral performance and associated metrics (e.g., PSS and JND) were monitored for the next 3 weeks. Ultimately, the first experimental series served to confirm that audiovisual temporal acuity in normal-hearing rats, like in humans, is influenced by sound intensity, as well as to provide additional context when interpreting any noise-induced changes in perceptual judgment caused by a permanent loss of auditory sensitivity.

## Materials and Methods

Overall, the present study included two experimental series: (1) to investigate how modulating sound intensity affects performance on either the TOJ task (Experiment 1A) or SJ task (Experiment 1B); and (2) to determine whether noise-induced hearing loss affected the perception of simultaneity (Experiment 2A; TOJ task) or synchrony (Experiment 2B; SJ task). A total of 31 adult male Sprague-Dawley rats (Charles River Laboratories Inc., Wilmington, MA, USA) were used in the present study: Experiment 1A (*n* = 10); Experiment 1B (*n* = 10); Experiment 2A (*n* = 9; one which was also used in Experiment 1A); Experiment 2B (*n* = 9; six of which were also used in Experiment 1B). This study was carried out in accordance with the recommendations of Canadian Council of Animal Care. The protocol was approved by the University of Western Ontario Animal Care and Use Committee.

### Behavioral Apparatus and Sensory Stimuli

**Figure 1:**
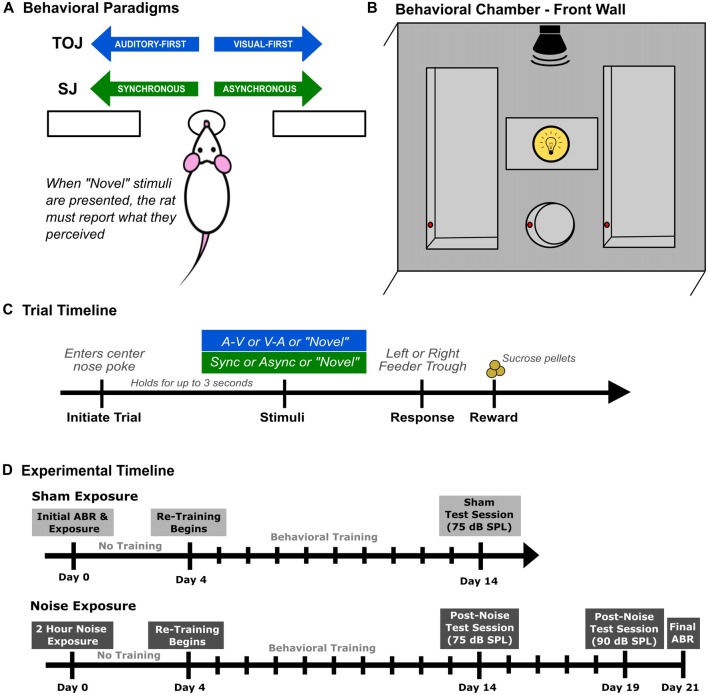
Rat audiovisual behavioral tasks and chamber set up. Rats were trained on either an audiovisual temporal order judgment (TOJ) task or a synchrony judgment (SJ) task. **(A)** Overview of both behavioral tasks. Through a series of stages, rats were trained using a two-alternative forced choice paradigm, where they were required to choose the right or left feeder trough depending on the stimulus condition presented. For example in the TOJ task, rats were trained to discriminate between auditory-first and visual-first trials, where the rats respond to the left feeder trough when an auditory-first stimulus condition is presented and the right feeder trough when a visual-first stimulus condition is presented. **(B)** Schematic of the front wall of the behavioral chamber used for both tasks. The front wall of the chamber consists of a left and right feeder trough and a center nose poke, all outfitted with infra-red (IR) detectors (represented by the red circles within the feeders and nose poke) used for response detection and trial initiation, respectively. The auditory stimulus was delivered from a speaker located above the center nose poke from above the chamber and the visual stimulus was presented from the LED located immediately above the center nose poke. **(C)** Representative timeline of a single trial for rats trained on either the audiovisual TOJ or SJ task. **(D)** The experimental timeline for the second experimental series consisting of two different test sessions completed after sham or noise exposure.

Behavioral training and testing were performed in a standard modular test chamber (ENV-008CT; Med Associates Inc., St. Albans, VT, USA) that was housed within a sound-attenuating box (29′ W by 23.5′ H by 23.5′ D; Med Associates Inc., St. Albans, VT, USA). The front wall of the behavioral chamber was equipped with a center nose poke, a left feeder trough and a right feeder trough that were each fitted with an infrared (IR) detector (see Figure 1B), whereas the back wall was equipped with a house light that illuminated the test chamber. Real-time processing hardware (RZ6 and BH-32, Tucker Davis Technologies, Alachua, FL, USA) were interfaced with the test chamber. Custom behavioral protocols running in Matlab (EPsych Toolbox^1^) monitored the nose poke responses, and controlled the presentation of the auditory and visual stimuli, as well as the positive reinforcement (i.e., sucrose pellet delivery) and punishment (i.e., turning off the house light and an inability to commence the next trial).

The visual stimulus was a 50 ms light flash (27 lux) from an LED (ENV-229M; Med Associates Inc., Fairfax, VT, USA) located above the center nose poke. The intensity of the visual stimulus was determined using a LED light meter (Model LT45, Extech Instruments, Nashua, NH, USA). The auditory stimulus was a 50 ms noise burst (1–32 kHz) from a speaker (FT28D, Fostex, Tokyo) mounted on the ceiling of the behavioral chamber near the front wall (see Figure 1B). Consistent with Schormans et al. (2017a), rats were trained on the behavioral tasks using a 75 dB sound pressure level (SPL) auditory stimulus. The auditory stimulus was calibrated using custom Matlab software with a 1/4-inch microphone (2530, Larson Davis) and preamplifier (2221; Larson Davis).

### Overview of Behavioral Training Procedures for the TOJ and SJ Tasks

Using appetitive operant conditioning, rats were trained on either an audiovisual TOJ task or an audiovisual SJ task which were both designed as two-alternative forced-choice paradigms. In the TOJ task, rats were trained to differentiate the temporal order of auditory and visual stimuli, whereas rats trained on the SJ task learned to differentiate between trials when the visual and auditory stimuli were presented synchronously or when the visual stimulus preceded the auditory stimulus. For both behavioral tasks, rats began training at 70 days old (body mass: 281 ± 4.7 g), and were trained 6 days a week. All experimental testing took place when the rats were between 6 months and 11 months of age.

Prior to commencing behavioral training, rats were weighed daily and maintained on a food restricted diet until they neared 85% of their free-feeding body mass. During the first few training sessions, unprompted nose pokes into the center port (which were detected by the IR beam; red circles in Figure 1B) resulted in the presentation of an audiovisual stimulus condition, and the delivery of a 45 mg sucrose pellet (Bio-Serv, Frenchtown, NJ, USA) to the feeder associated with the stimulus condition (i.e., TOJ task: auditory-first = left trough, visual-first = right trough; SJ task: synchronous = left trough, asynchronous = right trough; Figure 1A). Furthermore, rats were positively reinforced with a second pellet if they went to the correct feeder trough following the stimulus presentation (as monitored with the IR detector; Figure 1B). The second pellet was delivered in order to help the rats associate a given feeder trough with a specific audiovisual stimulus condition.

After three consecutive training sessions, the initial pellet reinforcement was eliminated, and now the delivery of a pellet was contingent on the rats selecting the correct feeder trough in response to a given stimulus condition. At this stage of the training procedure, the SOA was maintained at 400 ms. More specifically, in the TOJ task, rats were required to differentiate between “visual-first” and “auditory-first” conditions, where the timing between stimuli presented was 400 ms (i.e., the auditory stimulus was presented 400 ms prior to the visual stimulus and vice versa). Similarly, in the SJ task, rats were required to differentiate between synchronous (i.e., 0 ms SOA) and asynchronous audiovisual stimuli in which the visual stimulus preceded the auditory stimulus by 400 ms. Throughout all stages of the behavioral training procedure, sessions consisted of 30-min of daily training, where correct feeder trough responses were reinforced with a sucrose pellet, and incorrect responses resulted in the house light turning off for up to 15 s, during which time a new trial could not be initiated (Figure 1C). Consistent with previous investigations, the daily amount of food provided was adjusted so that each rat’s body mass increased with age, while providing enough motivation for it to complete ~200 trials in a session (Stolzberg et al., 2013; Schormans et al., 2017a).

In order for rats to move on to the next training stage, they were required to correctly discriminate between the two audiovisual stimulus conditions (i.e., TOJ task: auditory-first vs. visual-first; SJ task: synchronous vs. asynchronous) with >75% accuracy. Once this performance criterion was achieved for three consecutive days, the SOA timing was reduced to 300 ms for both stimulus conditions in the TOJ task, as well as the asynchronous stimulus condition in the SJ task. Consistent with the previous stage, rats trained for 30 min/day until the criterion of 75% correct was achieved for both stimulus conditions. Rats progressed to the final stage of training once they reached the 75% performance criterion in five consecutive days. During this final training stage, the SOA was reduced to 200 ms for both stimulus conditions in the TOJ task, as well as the asynchronous stimulus condition in the SJ task. The second stimulus condition in the SJ task (i.e., synchronous audiovisual stimuli) did not change throughout the training stages. As described in further detail below, each rat was considered ready to progress to experimental test days once it had achieved >80% accuracy for five consecutive days on the final training stage.

### Experiment 1—Modulation of Sound Intensity

#### Experiment 1A—TOJ Task Performance and Analysis

Once rats (*n* = 10) had successfully completed all stages of behavioral training for the TOJ task, experimental test sessions were introduced in which novel SOAs were presented to determine each rat’s audiovisual temporal order perception. Three different experimental tests were performed in each rat that differed in the intensity of the auditory stimulus (i.e., 60, 75 or 90 dB SPL). Experimental tests were randomized in order to counterbalance the potential influence of training duration. For each of the tests completed, seven SOAs were randomly delivered (i.e., 0, ±40, ±100 and ±200 ms); however, to reduce the potential of developing a side bias, 70% of the trials were the same as the training stimuli (i.e., TOJ task: ±200 ms SOA). The remaining 30% of trials consisted of the random presentation of the novel SOAs (0, ±40, ±100 ms). A sucrose pellet was delivered following each novel SOA regardless of whether a correct or incorrect response was made. In contrast, the trained stimulus conditions were positively reinforced for correct responses with sucrose pellets, and punished for incorrect responses with a 15-s timeout. Within a given test session, rats performed a minimum of 10 trials at each of the novel SOAs (mean of 13 ± 0.3 trials) to ensure that they had experienced a sufficient number of trials to accurately determine their ability to judge the relative timing of audiovisual stimuli (Schormans et al., 2017a).

To assess the effect of sound intensity on audiovisual temporal order perception, multiple metrics were extracted from each of the experimental test sessions. For all seven SOAs, performance was measured as the proportion of trials in which the rat perceived the stimuli as visual-first (i.e., responded to the right feeder trough, Figure 1A). Test sessions were repeated if the trained stimuli (i.e., ±200 ms) did not reach the criterion of 70% correct or if a strong side bias formed. Consistent with Vatakis et al. (2007), a psychophysical profile at each sound intensity was generated for each rat by plotting straight lines between each of the neighboring SOAs tested, and the associated slope and intercept values were calculated. Using these values, the PSS was calculated by determining the SOA at which 50% of the responses were perceived as visual-first. In addition, the JND was determined by taking the difference between the SOAs at which 25% and 75% of the responses were perceived as visual-first, and then dividing by two (Vroomen and Stekelenburg, 2011). The PSS and JND were calculated for each of the test sessions, and averaged across rats within a given sound intensity (i.e., 60, 75 and 90 dB SPL).

#### Experiment 1B—SJ Task Performance and Analysis

Once rats trained on the SJ task (*n* = 10) had successfully reached the final criterion (i.e., >80% correct on synchronous (0 ms SOA) and asynchronous (200 ms SOA) conditions for five consecutive days), experimental test sessions were completed that differed in the intensity of the auditory stimulus (i.e., 60, 75 or 90 dB SPL). Consistent with the TOJ task, experimental tests were randomized in order to counterbalance the potential influence of training duration. Test sessions consisted of the random presentation of five SOAs (i.e., the visual stimulus preceded the auditory stimulus by 0, 10, 40, 100 or 200 ms). On each of the test sessions, the trained stimulus conditions (i.e., 0 ms and 200 ms SOAs) made up 70% of the trials presented, and these trials continued to be reinforced with sucrose pellets for correct responses and punished with 15-s timeouts for incorrect responses. The remaining 30% of the trials were equally divided among the novel SOAs (i.e., 10, 40, and 100 ms SOAs), and were reinforced with a sucrose pellet regardless of whether a correct or incorrect response was made. Within a given test session, rats were presented a minimum of 18 trials at each of the novel SOAs (mean of 25 ± 0.5 trials); a suitable number of trials from which it was possible to accurately determine each rat’s perception of synchrony (Schormans et al., 2017a).

Ultimately, to assess the effect of sound intensity on audiovisual SJs, various metrics were extracted from each of the experimental test sessions. For all five SOAs, performance was measured as the proportion of trials in which the rat perceived the stimuli as synchronous (i.e., they responded to the left feeder trough, Figure 1A). Test sessions were repeated if the trained stimuli (i.e., 0 ms and 200 ms SOAs) did not reach the criterion of 70% correct or if a strong side bias formed. For each rat and a given sound intensity, a psychophysical profile was generated by plotting straight lines between each of the neighboring SOAs tested, and the associated slope and intercept values were tabulated. Using these calculated values, two audiovisual asynchrony thresholds (50% and 70%) were extracted in order to evaluate the perceptual consequences of sound intensity on the audiovisual SJ task. Thresholds of 50% and 70% were extracted as they are common values used to determine the TBW in humans (Baškent and Bazo, 2011; Stevenson and Wallace, 2013; Eg et al., 2015; Kaganovich, 2016).

### Experiment 2—Noise Exposure and Audiovisual Temporal Acuity

To determine how hearing loss affects audiovisual temporal acuity, rats that were trained on the TOJ task (*n* = 9; Experiment 2A) or SJ task (*n* = 9; Experiment 2B) underwent a sham and loud noise exposure, after which their behavioral performance during subsequent training and testing sessions were monitored for the next 3 weeks. As outlined in the experimental timeline (Figure 1D), once the rats had reached the training performance criterion, their baseline hearing sensitivity was assessed with an auditory brainstem response (ABR) prior to the 2-h sham exposure (Day 0). After a 3-day hiatus, rats returned to performing training sessions for 10 days, followed by a test session on Day 14 (see Figure 1D). Once the training and testing sessions were completed following the sham exposure, all rats underwent a 2-h noise exposure. Consistent with the sham exposure procedure, behavioral performance was monitored for 3 weeks following the noise exposure. In addition to the test session completed on Day 14, noise-exposed rats also performed a final test session on Day 19 during which time the intensity of the auditory stimulus was increased from the standard 75 dB SPL to 90 dB SPL. A final ABR was performed 3 weeks after the noise exposure (Day 21) to assess the level of permanent hearing loss. Because all trained rats first underwent a sham exposure (see Figure 1D), this allowed for a within-subject control of the possible effects of anesthesia and/or time delay before returning to the behavioral sessions post-noise exposure.

#### Hearing Assessment

Hearing sensitivity before and after noise exposure were assessed using an ABR, which was performed in a double-walled sound-attenuating chamber. Rats were anesthetized with ketamine (80 mg/kg; IP) and xylazine (5 mg/kg; IP), and subdermal electrodes were positioned at the vertex, over the right mastoid process and on the back. Throughout the procedure, body temperature was maintained at ~37°C using a homeothermic heating pad (507220F; Harvard Apparatus, Kent, UK). Auditory stimuli consisted of a click (0.1 ms) and two tones (4 kHz and 20 kHz; 5 ms duration and 1 ms rise/fall time) which were generated using a Tucker-Davis Technologies (TDT, Alachua, FL, USA) RZ6 processing module at 100 kHz sampling rate. Stimuli were delivered from a magnetic speaker (MF1; TDT) positioned 10 cm from the animal’s right ear. The left ear was occluded with a custom foam earplug. Each of the stimuli were presented 1,000 times (21 times/s) at decreasing intensities from 90 dB to 10 dB SPL in 10 dB SPL steps. Near threshold, successive steps were decreased to 5 dB SPL, and each level was presented twice in order to best determine ABR threshold using the criteria of just noticeable deflection of the averaged electrical activity within the 10 ms window (Popelar et al., 2008; Schormans et al., 2017b). The auditory evoked activity was collected using a low impedance headstage (RA4L1; TDT), preamplified and digitized (RA16SD Medusa preamp; TDT), and sent to a RZ6 processing module via a fiber optic cable. The signal was filtered (300–3,000 Hz) and averaged using BioSig software (TDT). Sound stimuli for the ABR and noise exposure were calibrated with custom Matlab software (The Mathworks, Natick, MA, USA) using a 1/4-inch microphone (2530; Larson Davis, Depew, NY, USA) and preamplifier (2221; Larson Davis).

#### Noise Exposure

Rats were anesthetized with ketamine (80 mg/kg; IP) and xylazine (5 mg/kg: IP), and placed on a homeothermic heating pad to maintain body temperature at ~37°C. Noise exposure consisted of a calibrated broadband noise (0.8–20 kHz) delivered bilaterally at 120 dB SPL for 2 h. The broadband noise was generated with TDT software and hardware (RPvdsEx; RZ6 module), and delivered by a super tweeter (T90A; Fostex, Tokyo, Japan) which was placed 10 cm in front of the rat. This noise exposure protocol was chosen as it is known to cause persistent changes at the level of the auditory cortex (Popelar et al., 2008) as well as to induce crossmodal plasticity within higher-order sensory cortices (Schormans et al., 2017b).

#### Behavioral Testing and Performance Post-noise Exposure

Consistent with the experimental parameters described above, the sham/noise-exposed rats performed test sessions that included both the novel and training SOAs for audiovisual stimuli during the TOJ task (i.e., 0, ±40, ±100 and ±200 ms; Experiment 2A) and SJ task (i.e., visual preceding auditory by 0, 10, 40, 100 or 200 ms; Experiment 2B). Ultimately, for both the TOJ and SJ tasks, the effect of noise-induced hearing loss on audiovisual temporal acuity was determined by comparing the sham vs. noise exposure performance for the SOAs on the training sessions of Day 4–13, as well as the audiovisual psychophysical curves generated on Day 14 (i.e., 75 dB SPL) and Day 19 (i.e., 90 dB SPL). Furthermore, the PSS and JND were calculated for rats that performed the TOJ task, and the results were compared between the sham and noise exposure conditions. Based on performance during the SJ task, it was possible to determine the effect of noise-induced hearing loss on the temporal window of integration by comparing the audiovisual asynchrony thresholds (50% and 70%) in rats post-sham vs. post-noise exposure.

### Statistics and Data Presentation

The statistical analyses performed in the present study included one- and two-way repeated-measures analysis of variance (rmANOVA), and paired samples *t*-tests, depending on the comparison of interest (see “Results” section for the details of each specific comparison). If Mauchly’s test of sphericity was violated within the repeated-measures ANOVA, the Greenhouse-Geisser correction was used. SPSS software (version 25, IBM Corporation, Armonk, NY, USA) was used for statistical analyses, and GraphPad Prism (GraphPad Software Inc., La Jolla, CA, USA) was used to plot the results. Data are presented as the mean values ± standard error of the mean (SEM).

## Results

### Experiment 1A—Modulation of Sound Intensity Shifted the Perception of Simultaneity During the TOJ Task

**Figure 2:**
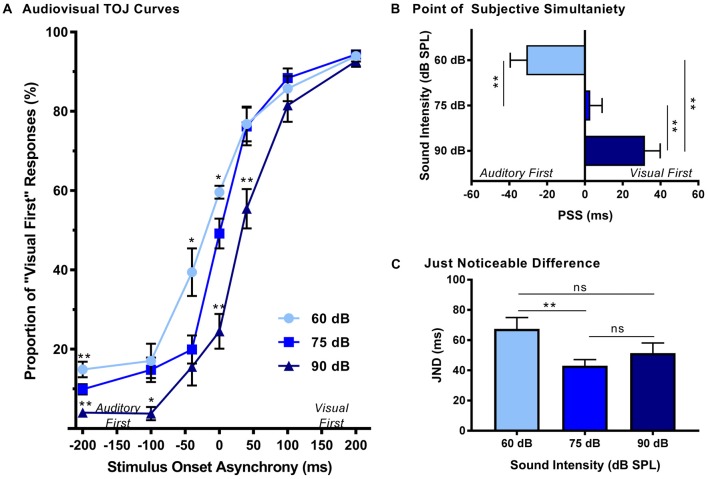
Effect of sound intensity on audiovisual temporal order perception. **(A)** Behavioral performance was plotted as the proportion of responses the rat perceived as “visual-first” (i.e., right feeder trough) for test days completed at 60 dB, 75 dB and 90 dB sound pressure level (SPL). A right-ward shift in the TOJ curve was observed as the intensity of the auditory stimulus increased. For example, at 0 ms stimulus onset asynchrony (SOA) there was an increase in “visual-first” responses at 60 dB SPL when compared to 75 dB SPL (*p < 0.01), and a significant decrease in “visual-first” responses at 90 dB SPL when compared to 75 dB SPL (**p < 0.001). **(B)** The point of subjective simultaneity (PSS) and **(C)** the just noticeable difference (JND) were derived from the TOJ task. For PSS, a significant difference was observed between all sound intensities (**p < 0.001), demonstrating a right-ward shift from “auditory-first” responses to “visual-first” responses as the sound intensity increased. For JND, a significance difference was only observed at the lowest sound intensity (i.e., 60 dB SPL), resulting in an increased window of integration (**p < 0.01, ns = not significant). Results are displayed as mean ± standard error of the mean (SEM), *n* = 10.

The effect of sound intensity on audiovisual temporal order perception was examined during the TOJ task using three testing conditions which differed in the intensity of the auditory stimulus presented (i.e., 60, 75 and 90 dB SPL). For each test session, the proportion of trials that were perceived as visual-first were determined for all SOAs ranging from −200 ms (i.e., auditory-first) to +200 ms (i.e., visual-first). Overall, a two-way rmANOVA revealed a significant interaction of sound intensity by SOA (*F*_(3.8,34.3)_ = 6.0, *p* < 0.01). To examine this interaction, *post hoc* paired samples *t*-tests were completed between the test sessions at 75 dB SPL and 60 or 90 dB SPL. As shown in Figure 2A, when performance was compared across all SOAs for 75 and 60 dB SPL testing conditions, a significantly higher proportion of trials were perceived as “visual-first” when the 60 dB SPL auditory stimulus was delivered 200 ms before the visual stimulus (*p* < 0.007). Although additional comparisons did not reach statistical significance once corrected for multiple comparisons (Bonferroni-adjusted *p*-value of 0.007), trends persisted at an SOA of −40 ms and 0 ms (see Table 1 for detailed statistics), in which the 60 dB SPL auditory stimulus was more likely to be perceived as visual-first (Figure 2A). Contrary to the results observed during the 60 dB SPL test session, as the sound intensity increased from 75 dB to 90 dB SPL, the majority of SOAs tested were predominantly perceived as auditory-first. More specifically, there was a significant decrease in the proportion of trials perceived as visual-first at SOAs of −200, 0, and 40 ms (*p* < 0.007; Figure 2A), demonstrating that the 90 dB SPL testing session influenced perception on both sides of simultaneity, whereas the 60 dB SPL session only affected auditory-first SOAs. Although additional comparisons did not reach statistical significance, the aforementioned results persisted as trends for the −100 ms SOA (see Table 1), in which the 90 dB SPL auditory stimulus was more likely to be perceived as auditory-first (Figure 2A). Taken together, these results demonstrate that sound intensity influenced the perception of audiovisual stimuli at various SOAs, with louder stimuli having the largest effect on judgments of audiovisual temporal order.

**Table 1:** Effect of auditory intensity and hearing loss on audiovisual temporal perception at all stimulus onset asynchronies (SOAs) when compared to 75 dB sound pressure level (SPL) testing sessions.

Experiment	SOA (ms)	*t*-score	*p*-value
**Experiment 1A**			
Decreased sound intensity	−200	4.29	2.02 × 10^−3^
(75 dB vs. 60 dB SPL)	−100	0.69	n.s.
	−40	3.29	9.37 × 10^−3^
	0	3.30	9.20 × 10^−3^
	40	−0.20	n.s.
	100	−0.87	n.s.
	200	−0.34	n.s.
**Experiment 1A**			
Increased sound intensity	−200	−5.05	6.95 × 10^−4^
(75 dB vs. 90 dB SPL)	−100	−3.04	1.40 × 10^−2^
	−40	−1.40	n.s.
	0	−5.28	5.08 × 10^−4^
	40	−4.21	2.28 × 10^−3^
	100	−1.72	n.s.
	200	−1.37	n.s.
**Experiment 2A**			
Increased sound intensity	−200	3.09	1.49 × 10^−2^
(Post-Noise: 75 dB vs. 90 dB SPL)	−100	0.61	n.s.
	−40	3.75	5.64 × 10^−3^
	0	3.09	1.49 × 10^−2^
	40	4.18	3.07 × 10^−3^
	100	−0.01	n.s.
	200	−0.36	n.s.

*n.s., not significant*.

In addition to the analyses completed on the TOJ psychophysical curves, the PSS and JND were calculated and compared across the three sound intensity testing conditions. As expected based on the TOJ psychophysical curves, a one-way rmANOVA revealed that sound intensity influenced the perception of audiovisual simultaneity (i.e., PSS; *F*_(2,18)_ = 36.7, *p* < 0.001). Consistent with our previous study (Schormans et al., 2017a), during the 75 dB SPL testing condition, the PSS was centered around an SOA of 0 ms (PSS = 2.7 ± 6.3 ms; Figure 2B). However, when the intensity of the auditory stimulus was decreased, the PSS also decreased (*p* < 0.001), such that the auditory stimulus needed to be presented well before the visual stimulus in order for the stimulus pair to be perceived as simultaneous (Figure 2B). The opposite pattern occurred when the intensity of the auditory stimulus was 90 dB SPL, as the PSS was significantly increased (*p* < 0.001). Interestingly, although the rats’ PSS was greatly affected by the intensity of the auditory stimulus, their ability to accurately discriminate the temporal order of the audiovisual stimuli (i.e., JND) was less affected (one-way rmANOVA, *F*_(2,18)_ = 5.0, *p* < 0.05). For example, whereas the testing condition with the 60 dB SPL auditory stimulus showed a significant increase in JND compared to 75 dB SPL, no other differences were observed (Figure 2C). Overall, these collective results demonstrate that sound intensity influenced the rats’ perception of simultaneity, but did not appreciably affect their sensitivity to reliably detect differences in the timing of the stimuli.

### Experiment 2A—Rapid Recalibration of Audiovisual Temporal Order Perception Following Hearing Loss

The effect of noise exposure on hearing sensitivity was assessed for rats trained on the TOJ task (*n* = 9) by comparing their ABR thresholds for the 4 kHz, 20 kHz and click stimuli pre- and post-noise exposure. A two-way rmANOVA (time × stimulus type) revealed a significant interaction of time by stimulus type (*F*_(2,16)_ = 7.26, *p* < 0.01). Overall, noise exposure increased ABR thresholds across all stimuli with the 20 kHz tone showing the greatest threshold shift (pre-noise: 20.6 ± 1.3 dB SPL vs. post-noise: 53.9 ± 5.2 dB SPL) compared to the 4 kHz tone (pre-noise: 28.9 ± 1.4 dB SPL vs. post-noise: 53.9 ± 4.6 dB SPL), and click stimulus (pre-noise: 26.1 ± 0.7 dB SPL vs. post-noise: 46.1 ± 3.2 dB SPL).

**Figure 3:**
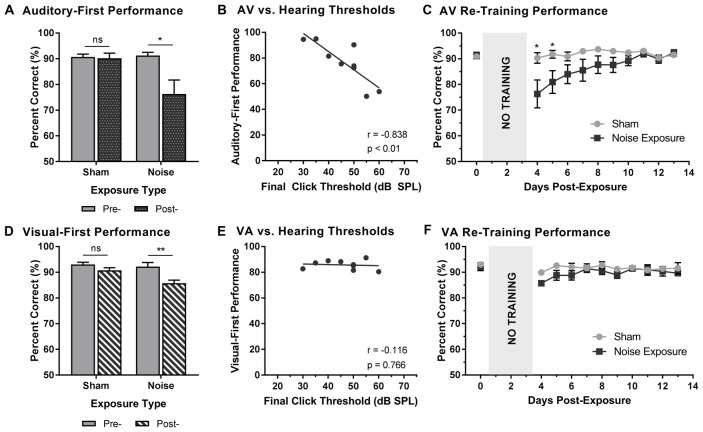
Altered auditory- and visual-first performance during TOJ training sessions following noise exposure. **(A)** Auditory-first performance and **(D)** visual-first performance pre- and 3 days post-exposure to a loud noise or sham. Following noise exposure there was a slight decrease in auditory-first performance (*p < 0.05, ns = not significant), as well as a significant decrease in visual-first performance (**p < 0.02, ns = not significant). Solid bars represent pre-exposure performance, and patterned bars represent post-exposure performance. Correlation results for **(B)** auditory-first performance and **(E)** visual-first performance as a function of final hearing sensitivity (i.e., click thresholds). Gray circles represent the individual data for each rat post-noise exposure. The solid line represents the linear regression line, and the Pearson correlation results along with the significance levels are displayed in the bottom of the panel. Behavioral performance on **(C)** auditory-first trials and **(F)** visual-first trials were monitored for 10 days post-exposure. A decrease in performance on auditory-first trials was observed following noise exposure during the first two training sessions (*p < 0.05). Results are displayed as mean ± SEM, *n* = 9.

Following a 3-day hiatus, rats that were trained on the TOJ task returned to the behavioral chamber for daily training sessions. As described above, training sessions consisted of the random presentation of auditory- or visual-first stimuli at an SOA of 200 ms. To determine the effect of hearing loss on judgments of audiovisual temporal order, performance on trials made up of auditory-first stimuli were analyzed pre- and post-exposure. A two-way rmANOVA (exposure × time) for auditory-first stimuli revealed a significant interaction of exposure by time (*F*_(1,8)_ = 8.6, *p* < 0.05). As can be seen in Figure 3A, a comparison of performance pre- and post-exposure showed a decrease in performance on auditory-first trials following noise exposure (*p* < 0.05; Figure 3A). Next, we investigated if there was a relationship between TOJ task performance and the degree of hearing loss. A Pearson correlational analysis revealed a significant relationship between final click thresholds and auditory-first performance 3 days following noise exposure (*r* = −0.84, *p* < 0.01), such that higher hearing thresholds (i.e., greater degree of hearing loss) resulted in the larger impairments in auditory-first performance (Figure 3B). Not surprisingly, following the sham exposure, there was no difference in performance on auditory-first trials (*p* = 0.80; Figure 3A). In addition to the first training session, performance was monitored over a total of 10 days post-exposure, at which point the first experimental test session was completed (i.e., post-exposure test at 75 dB SPL). A two-way rmANOVA revealed a significant interaction of exposure by training session (*F*_(2.7,21.4)_ = 4.0, *p* < 0.05), and *post hoc* paired samples *t*-tests demonstrated a slight decrease in auditory-first performance during the first two training sessions (i.e., Day 4 and 5; *p* < 0.05). Following the second training session (i.e., Day 5), performance returned to normal (i.e., equivalent to post-sham exposure performance, *p* > 0.05), indicating the auditory-first performance rapidly re-calibrated following adult-onset hearing loss (Figure 3C).

To further explore the effect of noise exposure on judgments of audiovisual temporal order, performance on visual-first trials was analyzed pre- and post-exposure. A two-way rmANOVA (exposure × time) revealed a significant interaction of exposure by time (*F*_(1,8)_ = 7.7, *p* < 0.05). Similar to the results during the auditory-first performance, there was a significant decrease in performance on visual-first trials following noise exposure (*p* < 0.01; Figure 3D). As expected, no difference was observed following the sham exposure (*p* = 0.13). Contrary to the auditory-first performance (Figure 3B), there was no significant relationship between final click thresholds and visual-first performance 3 days following noise exposure (Pearson correlational analysis; *r* = −0.01, *p* = 0.76; Figure 3E). Moreover, visual-first performance showed no impairments over the course of the 10 days post-exposure, as there was no effect of training session (*F*_(3.4,27.5)_ = 2.3, *p* = 0.09) and no interaction of training session by exposure (*F*_(3.8,30.5)_ = 1.1, *p* = 0.38; Figure 3F). Taken together, these results demonstrate that hearing loss predominantly influenced performance on trials when the auditory stimulus was presented before the visual stimulus.

### Experiment 2A—Audiovisual Temporal Order Perception in Noise-Exposed Rats Remained Sensitive to Sound Intensity Modulation

**Figure 4:**
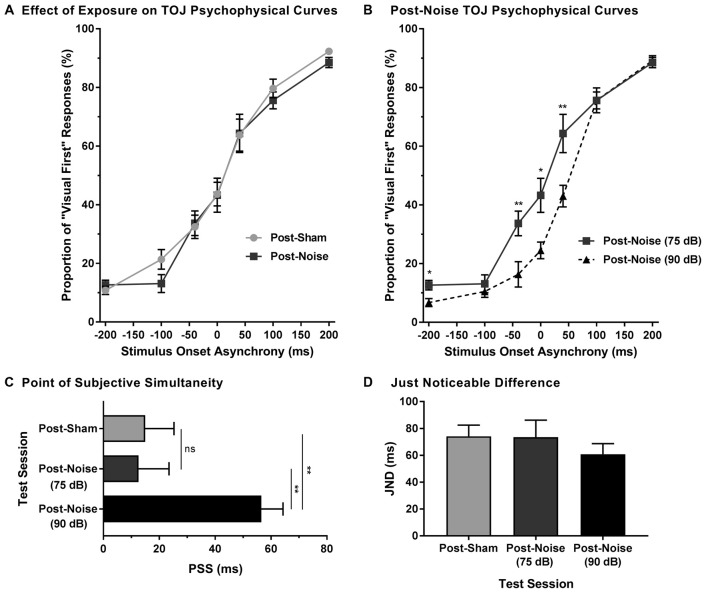
Preserved audiovisual temporal perception following adult-onset hearing loss. **(A)** Test sessions at 75 dB SPL were completed 2 weeks following exposure to a loud noise (i.e., post-noise) or quiet (i.e., post-sham). **(B)** An additional test session was completed at 90 dB SPL (i.e., post-noise (90 dB SPL)) and compared to the test session at 75 dB SPL (i.e., post-noise (75 dB SPL)), in order to determine if temporal perception remained sensitive to sound intensity. For all test sessions, performance was plotted as the proportion of trials that the rats perceived as “visual-first” (i.e., responded to the right feeder trough; *p < 0.05, **p < 0.007). **(C)** The PSS and **(D)** the JND were derived from each of the test sessions (**p < 0.01, ns = not significant). Results are displayed as mean ± SEM, *n* = 9.

To examine the effect of noise-induced hearing loss on audiovisual temporal perception, experimental tests were completed 2 weeks following sham exposure and noise exposure. Consistent with Experiment 1A, for each test session, the proportion of trials that were perceived as visual-first were calculated for all SOAs. A two-way rmANOVA (exposure × SOA) revealed a main effect of SOA (*F*_(2.3,18.1)_ = 190.5, *p* < 0.001) and no effect of exposure (*F*_(1,8)_ = 0.25, *p* = 0.634), as well as no interaction of exposure by time (*F*_(6,48)_ = 0.43, *p* = 0.859). Thus, despite an initial difficulty in differentiating the temporal order of audiovisual stimuli in the first few days following noise exposure (Figures 3A,C), the ability to accurately judge the temporal order of audiovisual stimuli returned to pre-exposure performance levels in rats with permanent hearing loss (Figure 4A).

To determine whether audiovisual temporal perception continued to be sensitive to changes in sound intensity following hearing loss, an additional experimental test session was conducted in which the intensity of the auditory stimulus was increased to 90 dB SPL. A two-way rmANOVA revealed a significant interaction of sound intensity by SOA (*F*_(6,48)_ = 5.7, *p* < 0.001). As shown in Figure 4B, when performance was compared across all SOAs at 75 and 90 dB SPL post-noise testing conditions, a significantly higher proportion of trials were perceived as “auditory-first” when the 90 dB SPL auditory stimulus was delivered at an SOA of −40 ms and 40 ms (*p* < 0.007). Although additional comparisons did not reach statistical significance once corrected for multiple comparisons, trends persisted at an SOA of −200 ms and 0 ms, in which the 90 dB SPL auditory stimulus was more likely to be perceived as auditory-first (see Table 1). Thus, adult-onset hearing loss does not seem to impair audiovisual temporal perception, as the behavioral performance of the noise-exposed rats remained sensitive to modulation of the intensity of the auditory stimulus.

Finally, to further examine the effect of hearing loss on judgments of audiovisual temporal order, perceived simultaneity (i.e., PSS) and temporal sensitivity (i.e., JND) were analyzed and compared across all experimental test sessions. Overall, we found that the PSS was indeed influenced by the experimental test session (one-way rmANOVA; *F*_(2,16)_ = 8.9, *p* < 0.01). Consistent with the results in the TOJ curves, PSS did not change following noise exposure (*p* = 0.87). However, when the sound intensity was increased from 75 dB to 90 dB SPL, the PSS of the noise-exposed rats significantly increased (*p* < 0.01; Figure 4C); results which were consistent with those observed in rats with normal hearing (Experiment 1A; Figure 1A). As can be seen in Figure 4D, JND did not differ across the various experimental test sessions (one-way rmANOVA; *F*_(2,16)_ = 1.3, *p* = 0.302). Overall, these results demonstrate that adult-onset hearing loss did not alter the perception of audiovisual simultaneity or temporal sensitivity as assessed with the TOJ task.

### Experiment 1B—Modulation of Sound Intensity Altered the Detection of Asynchronous Stimuli During the SJ Task

**Figure 5:**
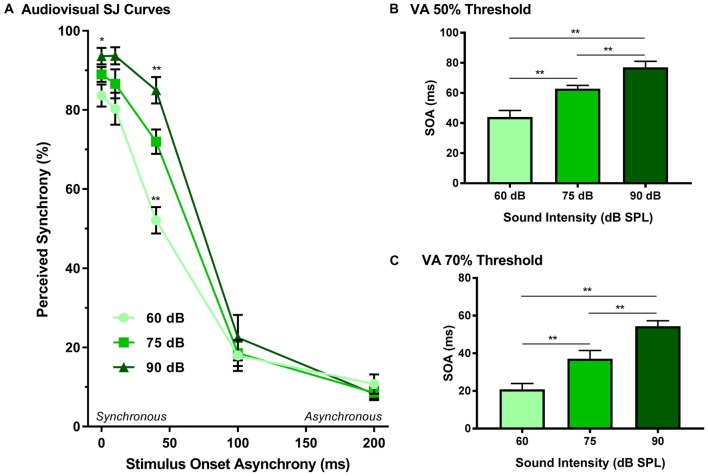
Effect of sound intensity on audiovisual synchrony perception as measured during an SJ task. **(A)** Behavioral performance was plotted as the proportion of trials the rat perceived as “synchronous” (i.e., left feeder trough) for tests completed at 60 dB, 75 dB and 90 dB SPL. A significant difference was observed at both 60 dB SPL (52.1 ± 3.3%) and 90 dB SPL (85.0 ± 3.3%) when compared to 75 dB SPL (69.0 ± 1.7%; **p < 0.01), indicating that as sound intensity increased, the rate of perceived synchrony also increased when the SOA was less than 100 ms (*p < 0.05). The **(B)** 50% threshold and **(C)** 70% threshold were derived from the SJ task. Consistent with the SJ curves, both thresholds showed a significant increase as the intensity of the auditory stimulus increased (**p < 0.01). Results are displayed as mean ± SEM, *n* = 10.

The effect of sound intensity on audiovisual synchrony perception was investigated during the SJ task using three testing conditions which differed in the intensity of the auditory stimulus presented (i.e., 60, 75 and 90 dB SPL). For each testing condition, the proportion of trials that were perceived as synchronous were determined for all SOAs ranging from 0 ms (i.e., synchronous) to 200 ms (i.e., asynchronous). Overall, a two-way rmANOVA revealed a significant interaction of sound intensity by SOA (*F*_(8,72)_ = 8.1, *p* < 0.001). To further investigate this interaction, *post hoc* paired samples *t*-tests were completed between the test sessions at 75 dB SPL and 60 or 90 dB SPL. As shown in Figure 5A, a comparison of performance across the various SOAs for the 75 and 60 dB SPL testing conditions revealed that the rats perceived a significantly lower proportion of trials as synchronous when the 60 dB SPL auditory stimulus was delivered 40 ms before the visual stimulus (*p* < 0.001). Consistent with the nature of these differences observed at 60 dB SPL, when the auditory stimulus intensity was increased from 75 dB to 90 dB SPL, there was a significant increase in the proportion of trials at an SOA of 40 ms that the rats perceived as synchronous (*p* < 0.008; see Table 2 for detailed statistics). Given that there were no performance differences when the visual stimulus preceded the various auditory stimuli by 100 or 200 ms (Figure 3A), the collective results show that modulation of sound intensity had the greatest effect on audiovisual synchrony perception when the pair of stimuli were presented relatively close together in time (0–100 ms).

**Table 2:** Effect of auditory intensity and hearing loss on audiovisual synchrony perception at all SOAs when compared to 75 dB SPL testing sessions.

Experiment	SOA (ms)	*t*-score	*p*-value
**Experiment 1B**			
Decreased sound intensity	0	−1.97	n.s.
(75 dB vs. 60 dB SPL)	10	−1.89	n.s.
	40	−4.81	9.63 × 10^−4^
	1000	−0.20	n.s.
	200	0.87	n.s.
**Experiment 1B**			
Increased sound intensity	0	2.43	3.78 × 10^−2^
(75 dB vs. 90 dB SPL)	10	2.10	n.s.
	40	4.36	1.82 × 10^−3^
	1000	1.41	n.s.
	200	−0.20	n.s.
**Experiment 2B**			
Post-Exposure at 75 dB SPL	0	0.89	n.s.
(Post-Sham vs. Post-Noise)	10	2.53	3.53 × 10^−2^
	40	−1.31	n.s.
	1000	−3.99	4.03 × 10^−3^
	200	−2.57	3.33 × 10^−2^
**Experiment 2B**			
Increased sound intensity	0	−1.63	n.s.
(Post-Noise: 75 dB vs. 90 dB SPL)	10	−2.16	n.s.
	40	−4.65	1.64 × 10^−3^
	1000	0.26	n.s.
	200	1.05	n.s.

*n.s., not significant*.

In addition to analyzing the role of sound intensity modulation on the SJ psychophysical curves, the 50% and 70% audiovisual asynchrony thresholds were extracted and compared across all sound intensities, as these thresholds represent criteria used previously to determine the TBW (Stevenson and Wallace, 2013). A one-way rmANOVA revealed a significant main effect of sound intensity for the 50% threshold (*F*_(2,18)_ = 44.2, *p* < 0.001), whereby the rats’ threshold significantly increased (*p* < 0.001) in accordance with the intensity of the auditory stimulus (Figure 5B). Similarly, a significant main effect was also observed at the 70% threshold (one-way rmANOVA; *F*_(1.2,11.0)_ = 30.1, *p* < 0.001), such that when the auditory stimulus intensity increased from 60 dB to 90 dB SPL, there was a significant widening of the right-sided TBW (Figure 5C). Thus, these collective results indicate that the louder the sound intensity during a flash-beep SJ task, the longer the time interval that was needed between the visual and auditory stimuli for the rats to correctly judge that the stimulus pair was indeed asynchronous.

### Experiment 2B—Persistent Impairments in the Ability to Judge the Synchrony of Audiovisual Stimuli Following Adult-Onset Hearing Loss

Alterations in hearing sensitivity were assessed pre- and post-exposure for the rats trained on the SJ task (*n* = 9) by comparing their ABR thresholds for the 4 kHz, 20 kHz and click stimuli. As expected, a two-way rmANOVA revealed a significant interaction of time by stimulus type (*F*_(2,16)_ = 11.2, *p* < 0.01). Moreover, Bonferroni corrected *post hoc* *t*-test revealed that noise exposure caused a significant increase in the ABR threshold of the click (pre-noise: 26.7 ± 0.8 dB SPL vs. post-noise: 54.4 ± 3.7 dB SPL, *p* < 0.001), 4 kHz (pre-noise: 28.3 ± 1.2 dB SPL vs. post-noise: 61.7 ± 3.3 dB SPL, *p* < 0.001), and 20 kHz tone (pre-noise: 23.3 ± 0.8 dB SPL vs. post-noise: 63.9 ± 4.5 dB SPL, *p* < 0.001).

**Figure 6:**
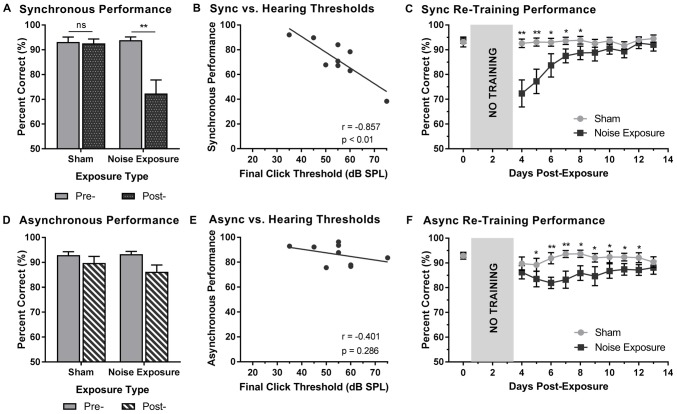
Hearing loss impaired performance during SJ training sessions. Performance on **(A)** synchronous and **(D)** asynchronous trials was compared pre- and 3 days post- exposure to a loud noise or sham. Following noise exposure, a significant decrease in performance on synchronous trials was observed (**p < 0.02, ns = not significant). No difference was observed on asynchronous trials. Solid bars represent pre-exposure performance and patterned bars represent post-exposure performance. Correlation results for **(B)** synchrony performance and **(E)** asynchrony performance were plotted as a function of final hearing sensitivity (i.e., click thresholds). Gray circles represent the individual data for each rat post-noise exposure. The solid line represents the linear regression line, and the Pearson correlation results along with the significance levels are displayed in the bottom of the panel. Behavioral performance on **(C)** synchronous and **(F)** asynchronous trials were monitored for 10 days following sham and noise exposure. Performance on synchronous trials returned to typical performance within 5 days, whereas performance on asynchronous trials remained consistently impaired across the majority of the training days (*p < 0.05, **p < 0.004). Results are displayed as mean ± SEM, *n* = 9.

Rats that were trained on the SJ task returned to daily behavioral training sessions 3 days following exposure to a loud noise or sham. Training sessions consisted of the random presentation of synchronous (i.e., 0 ms SOA) and asynchronous (i.e., 200 ms SOA) audiovisual stimuli. To examine the effect of hearing loss on the ability to accurately perceive the synchrony of audiovisual stimuli, performance on trials made up of synchronous and asynchronous stimuli were analyzed pre- and post-exposure. For synchronous stimuli, a two-way rmANOVA revealed a significant interaction of exposure by time (*F*_(1,8)_ = 15.0, *p* < 0.01). As can be seen in Figure 6A, exposure to the loud noise caused a significant decrease in performance on synchronous trials (*p* < 0.01). As expected, there was no change in performance on synchronous trials following the sham exposure (*p* = 0.762). Next, we examined the rats’ performance on synchronous trials following noise exposure to determine if this performance correlated with final hearing thresholds. Indeed, a Pearson correlational analysis revealed a significant negative relationship between final click thresholds and synchronous performance 3 days following noise exposure (*r* = −0.857, *p* < 0.01; Figure 6B). Therefore, the perceptual ability of noise-exposed rats to judge the synchrony of the audiovisual stimuli was dependent on their level of hearing impairment; a higher proportion of trials were perceived to be asynchronous if the rats had a greater degree of hearing impairment.

Beyond assessing performance in the first training session following the noise exposure, synchrony perception was also monitored for 10 days, after which the first experimental test session was completed (i.e., post-exposure test at 75 dB SPL). A two-way rmANOVA revealed a significant interaction of exposure by training session (*F*_(3.2,25.6)_ = 7.9, *p* < 0.001). As shown in Figure 6C, a significant decrease in performance occurred during the first two training sessions (i.e., Day 4 and 5). While no other training sessions reached statistical significance once corrected for multiple comparisons (Bonferroni-adjusted *p-value* of 0.004), trends persisted on days 6 through 8 (*p* < 0.05), in which synchronous trials were more likely to be perceived as asynchronous. However, following the fifth training session (i.e., Day 8), performance returned to normal (i.e., equivalent to post-sham exposure performance, *p* > 0.05), suggesting that the ability to detect synchronous stimuli eventually recovered after noise exposure.

To further examine the effect of hearing loss on judgments of synchrony, performance on asynchronous trials during the first training session was also examined pre- and post-exposure. Surprisingly, a two-way rmANOVA only revealed a main effect for exposure (*F*_(1,8)_ = 6.6, *p* < 0.05); there was no effect of time (*F*_(1,8)_ = 2.6, *p* = 0.15) and no significant interaction of exposure by time (*F*_(1,8)_ = 1.3, *p* = 0.28). Therefore, contrary to synchronous trials (i.e., 0 ms SOA), the ability to categorize asynchronous trials (i.e., 200 ms SOA) was not influenced by exposure to a loud noise or sham (Figure 6D). Consistent with the analyses described above, asynchronous performance and final hearing thresholds were examined in order to determine if performance was dependent upon hearing sensitivity. A Pearson correlation analysis revealed no significant relationship between performance on asynchronous trials and final click thresholds (*r* = −0.4, *p* = 0.286). While performance on the first training session was relatively maintained (see Figure 6D), performance across the 10 training sessions prior to the first experimental test session was consistently impaired (Figure 6F). A two-way rmANOVA revealed a significant interaction of exposure by training session (*F*_(3.8,30.3)_ = 3.5, *p* < 0.05). A further examination of this interaction demonstrated significant impairments in performance on Day 6 and 7 (*p* < 0.004) as well as slight impairments on Day 5 and 8 through 13 (*p* < 0.05). Therefore, hearing loss caused persistent impairments in asynchrony detection, such that a greater proportion of trials were perceived as synchronous; findings which could ultimately relate to an impaired perceptual binding of audiovisual stimuli.

### Experiment 2B—Impairments in Asynchrony Detection Resulted in Altered Perceptual Binding of Audiovisual Stimuli Following Hearing Loss

**Figure 7:**
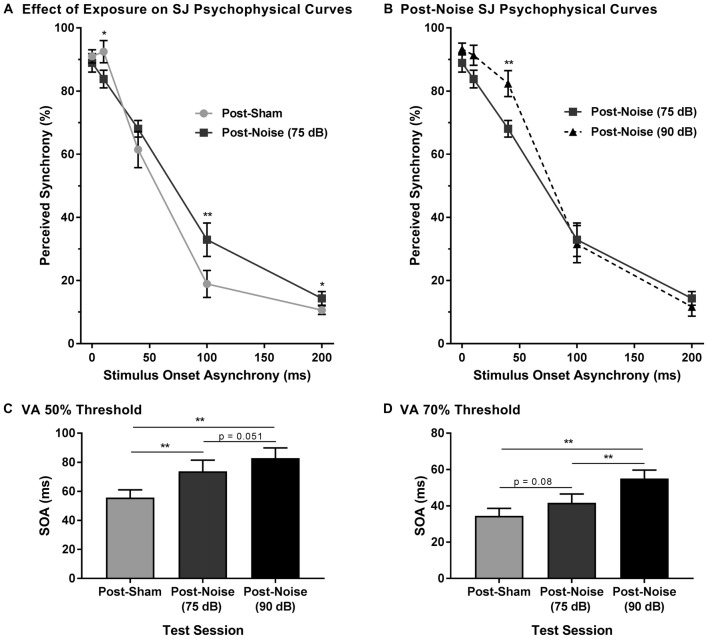
Impaired audiovisual synchrony perception following adult-onset hearing loss. **(A)** Experimental test sessions for the SJ task at 75 dB SPL were completed 2 weeks following exposure to a loud noise (i.e., post-noise) or quiet (post-sham). **(B)** An additional test session was completed at 90 dB SPL (i.e., post-noise (90 dB SPL)) and compared to the test session at 75 dB SPL (i.e., post-noise (75 dB SPL)), in order to determine if synchrony perception remained sensitive to sound intensity (*p < 0.05, **p < 0.01). For all test sessions, performance was plotted as the proportion of trials that the rats perceived as “synchronous” (i.e., responded to the left feeder trough). The **(C)** 50% threshold and **(D)** 70% threshold were derived from all SJ test sessions. Two weeks following noise exposure, there was a significant increase in the 50% threshold (**p < 0.017), and a modest increase in the 70% threshold (*p* = 0.08), indicative of a wider window of perceptual binding. Results are displayed as mean ± SEM, *n* = 9.

To explore the consequences of adult-onset hearing loss on audiovisual synchrony perception, rats trained on the SJ task were tested 2 weeks following exposure to a loud noise. For each test session, the rate of perceived synchrony was calculated as the proportion of trials that were perceived as synchronous for all SOAs ranging from 0 ms (i.e., synchronous) to 200 ms (i.e., asynchronous). A two-way rmANOVA revealed a significant interaction of exposure (i.e., post-sham vs. post-noise) by SOA (*F*_(2.1,16.6)_ = 6.9, *p* < 0.01). To further examine this interaction, *post hoc* paired samples *t*-tests completed between the two post-exposure test sessions (i.e., post-sham vs. post-noise) revealed that rats reported a significantly higher proportion of trials as synchronous following noise exposure when the visual stimulus was delivered 100 ms before the auditory stimulus (*p* < 0.01; Figure 7A). Although additional comparisons did not reach statistical significance once corrected for multiple comparisons, modest changes were observed at an SOA of 10 ms and 200 ms (see Table 2 for detailed statistics). Overall, these results demonstrate that adult-onset hearing loss impairs synchrony perception, such that truly asynchronous audiovisual stimuli were more likely to be perceived as synchronous.

To determine whether sound intensity was still capable of influencing synchrony perception following adult-onset noise-induced hearing loss, an additional test session was completed in which the intensity of the auditory stimulus was increased from 75 dB to 90 dB SPL. As predicted, a two-way rmANOVA revealed a significant interaction of sound intensity (i.e., 75 dB vs. 90 dB SPL post-noise) by SOA (*F*_(1.6,13.0)_ = 4.3, *p* < 0.05). Similar to the differences observed in normal-hearing rats in Experiment 1B, when the intensity of the auditory stimulus was increased from 75 dB to 90 dB SPL, noise-exposed rats showed a significant increase in the proportion of trials perceived as synchronous at an SOA of 40 ms (Figure 7B). Thus, audiovisual synchrony perception remained sensitive to changes in the intensity of the auditory stimulus, despite these same rats showing an impaired ability to detect asynchronous stimuli.

In addition to the analyses completed on the SJ psychophysical curves following hearing loss, the 50% and 70% audiovisual asynchrony thresholds were compared across all test sessions. Separate one-way rmANOVAs revealed a significant effect of test session for the 50% threshold (*F*_(2,16)_ = 14.3, *p* < 0.001) and the 70% threshold (*F*_(2,16)_ = 12.4, *p* < 0.01). As shown in Figure 7C, the 50% asynchrony threshold significantly increased following noise exposure (*p* < 0.01); findings indicative of a greater degree of temporal tolerance which could result in a broadened TBW. While the 70% threshold did not significantly increase following a noise exposure, a trend towards an increase in threshold was observed (*p* = 0.08; Figure 7D). Overall, despite this increase in the epoch of time over which the audiovisual stimuli appeared to be perceptually bound, the noise-exposed rats remained sensitive to changes in the intensity of the auditory stimulus; i.e., when the intensity of the auditory stimulus was increased, there was a significant increase in the 70% threshold (*p* < 0.01), as well as a trend towards an increase in the 50% threshold (*p* = 0.051). Thus, the collective results demonstrate that adult-onset hearing loss alters the perception of audiovisual synchrony.

## Discussion

To our knowledge, the present study represents the first comprehensive investigation into the degree to which audiovisual temporal acuity is influenced by adult-onset hearing loss, with a specific focus on the time-course of perceptual changes following loud noise exposure. Using operant conditioning, rats were trained and tested on either a TOJ task in which they reported the relative timing of audiovisual stimuli presented at various SOAs, or an SJ task in which they reported whether audiovisual stimuli were presented at the same moment in time or at different times. Ultimately, adult-onset hearing loss caused a differential effect on audiovisual temporal acuity depending on whether perception was assessed with the TOJ or SJ task. For example, performance on the TOJ task revealed that the perception of temporal order rapidly recalibrated following noise exposure, resulting in a preservation of temporal sensitivity. In contrast, noise-exposed rats showed a persistent impairment in their ability to detect asynchronous audiovisual stimuli during the SJ task, resulting in a greater tolerance of asynchronous stimuli which could manifest as a widening of their TBW. Taken together, these results provide important insight into the nature and extent of behavioral plasticity of audiovisual perception following adult-onset hearing loss.

### Stimulus Intensity Predicts Audiovisual Temporal Acuity

Prior to conducting our studies into the effect of adult-onset hearing loss on audiovisual temporal acuity, psychophysical testing was completed in normal-hearing rats for both the TOJ and SJ tasks in which the intensity of the auditory stimulus was modulated, while the intensity of the visual stimulus was held constant. Overall, the results of this first series of experiments demonstrated that sound intensity predicted audiovisual perception, such that when a lower-intensity sound was presented the rats were biased to perceive the audiovisual stimuli as asynchronous (SJ task), or as though the visual stimulus was presented first (TOJ task). As discussed below, these results are consistent with previous studies on humans that assessed PSS during TOJ tasks (Smith, 1933; Neumann et al., 1992; Neumann and Niepel, 2004; Boenke et al., 2009). For example, Boenke et al. (2009) found that increasing the intensity of the visual stimulus during a TOJ task caused the participants’ perception of simultaneity (i.e., PSS) to decrease; findings consistent to when we *lowered* the intensity of the auditory stimulus in the present study. Indeed, we found that when the sound intensity was lowered, the PSS was more likely to be perceived as “auditory-first” and conversely, when the sound intensity was increased, the PSS shifted to being perceived as “visual-first” (Figure 2B). Collectively, the results in humans and rats confirm that when the auditory or visual stimulus intensity is modulated, a predicable perceptual shift occurs regarding which stimulus modality was thought to have been presented first.

Previous studies that screened for synchrony perception using SJ tasks have demonstrated differential results when the intensity of both stimuli were modulated, perhaps due to different task parameters. For example, Smith (1933) observed minimal effects of stimulus intensity on participants’ perceptual judgment when presenting audiovisual stimuli on both sides of simultaneity. However, when Krueger Fister et al. (2016) presented stimuli only on the right-side of simultaneity (i.e., a flash-beep task with visual-first asynchronies), they observed that pairing weak auditory and visual stimuli resulted in a decreased ability to accurately perceive when the stimuli were asynchronous. Interestingly, using the same task parameters as Krueger Fister et al. (2016), we found that decreasing the intensity of only the auditory stimulus increased the proportion of trials reported as asynchronous, indicating that the rats exhibited an improvement in asynchrony detection during the SJ task. Thus, it appears that decreasing the intensity of both modalities increases the temporal offsets over which perceptual binding occurs (i.e., TBW widens), yet decreasing the intensity of only the auditory stimulus, potentially narrows the TBW. While the degree of temporal tolerance appears to move in opposite directions depending on whether the intensity of both modalities or a single modality are modulated, these collective results are in accordance with perceptual latencies. For example, stimuli that are of lower intensity tend to occur at a greater distance from the individual and thus result in greater temporal differences between the respective sensory receptors. Therefore, it has been postulated that the brain must compensate for lower stimulus intensities by providing a greater degree of tolerance, allowing for stimuli to be perceptually bound (Krueger Fister et al., 2016). However, when only a single stimulus is modulated, the intensity disparity between the two stimuli could result in a lower degree of temporal integration as the brain may be less likely to bind the stimuli because they are more likely perceived as two separate events. As the present study and that of Krueger Fister et al. (2016) used an SJ task that only presented stimuli on the right-side of simultaneity, further studies will be needed to determine how alterations in stimulus intensity influence the entire temporal window of integration. Ultimately, the collective results of the first experimental series complement our understanding of the factors that influence audiovisual temporal acuity, and may offer important considerations when interpreting TOJ and SJ task performance of participants with altered hearing sensitivity (e.g., those with hearing loss, or individuals who experience hyper-sensitivity to sounds).

### Hearing Loss and Audiovisual Temporal Acuity

Given that hearing loss reduces one’s sensitivity to environmental sounds, and we and others have shown that varying the intensity of an auditory stimulus alters perceptual judgments in normal-hearing participants, we reasoned that noise-induced hearing loss in adulthood may impact audiovisual temporal acuity. Interestingly, we found that 2–3 weeks after noise exposure rats with permanent hearing loss maintained their ability to judge the temporal order of the audiovisual stimuli, as PSS was unchanged, and their temporal sensitivity was preserved (i.e., JND was consistent). To our knowledge, this is the first investigation of the effect of hearing loss on audiovisual temporal perception as assessed with a TOJ task. That said, Baškent and Bazo (2011) used an SJ task to study individuals with a hearing impairment, and found that their level of perceptual binding (as assessed via the TBW) was similar to normal-hearing participants; findings that disagree with the persistent impairment in asynchrony detection ability observed in the present study. However, these conflicting results could arise due to experimental differences, including the age of the participants used in each of the experimental groups, the duration of hearing loss (2–3 weeks in rats vs. 6–28 years in humans), as well as the absolute/relative intensity of the auditory stimuli used in the SJ tasks (75 or 90 dB SPL in rats vs. adjusted to compensate for sensation level in each hearing-impaired participant). The presentation of auditory stimuli at sensation level (i.e., adjusted based on the degree of hearing loss in each participant) is a particularly important experimental difference, as stimulus intensity is known to have a significant influence on audiovisual perception. Thus, future studies in subjects with hearing-impairments should include psychophysical testing at both an absolute auditory intensity as well as at sensation level.

In considering the differential effects of hearing loss on the TOJ and SJ task performance observed in the present study, it is worth noting that previous research on normal-hearing participants has also shown disparate results between the two tasks. These differences in task performance are thought to arise partially from participant response biases and experimental methodology (Vatakis and Spence, 2007; Vatakis et al., 2008b; Vroomen and Keetels, 2010; García-Pérez and Alcalá-Quintana, 2012), or alternatively, because the TOJ and SJ task rely on distinct perceptual processes (Kostaki and Vatakis, 2018). Indeed, Zampini et al. (2003) suggested that the TOJ task performance may reflect processes related to temporal discrimination, whereas SJ tasks may be more related to temporal binding mechanisms. Examining our results under this proposed framework, it seems that temporal order perception is preserved, whereas the perceptual binding of stimuli is impaired following adult-onset hearing loss. Interestingly, a previous study found the opposite relationship in older participants (with corrected-to-normal hearing), who showed more difficulty in discriminating the temporal order of the auditory and visual stimuli, but their TBW during the SJ task was not different from younger adults (Bedard and Barnett-Cowan, 2016).

### Behavioral Plasticity of Audiovisual Temporal Acuity Following Adult-Onset Hearing Loss

Although we observed no effect of hearing loss on the TOJ task performance 2–3 weeks post-noise exposure, when the rats first resumed training on the task 3 days after noise exposure, they did show an impaired ability to accurately judge the temporal order of audiovisual stimuli when the auditory stimulus was presented before the visual stimulus. Moreover, this impairment on “auditory-first” trials was related to their level of hearing loss, such that the rats with the greatest hearing loss performed the poorest on the “auditory-first” trials. It was during the next 10 days of training that we observed a progressive shift in the rats’ perception of temporal order toward pre-noise exposure performance. Similar findings were observed for rats’ performing the SJ task, in which their ability to detect synchronous audiovisual stimuli was initially impaired in relation to the level of hearing loss, but this ability recovered progressively over the next 10 days. Overall, the daily exposure to the training stimuli pairings (e.g., TOJ task: ±200 ms SOA; SJ task: 0 and 200 ms SOA) may have resulted in the rats re-learning the association between the stimuli pairings within their new perceptual state (i.e., impaired hearing sensitivity from hearing loss), which ultimately led to a perceptual recalibration of audiovisual perception. Support for this suggestion comes from previous studies on normal-hearing participants which found that engagement in perceptual training paradigms that included trial-by-trial feedback (like in the present study) led to an improved ability to detect asynchronous audiovisual stimuli, thus resulting in a narrower temporal window of integration (Powers et al., 2009; De Niear et al., 2016, 2018). Future studies are needed to determine whether exposure to training stimuli is necessary for the preservation of audiovisual perception.

At this time, it remains uncertain why the perception of audiovisual temporal order fully recovered post-noise exposure, whereas there was a persistent impairment in the rats’ ability to detect asynchronous audiovisual stimuli during the SJ task. Given that aspects of the SJ task performance (i.e., synchrony detection) did indeed recover, it is reasonable to question whether it would just have required a longer duration (>3 weeks) for asynchrony detection and perceptual binding to also fully recalibrate following permanent hearing loss. In support of this possibility, Baškent and Bazo (2011) observed that participants with a relatively short duration of deafness had wider TBWs, which could suggest that, following auditory deprivation, synchrony perception may improve over time. Ultimately, based on the differential rates of recalibration post-noise exposure of the aforementioned features of audiovisual temporal acuity (e.g., PSS and JND from the TOJ task; synchrony/asynchrony detection and TBW from the SJ task), our collective results provide additional support for the suggestion that different perceptual processes likely underlie TOJ and SJ task performance.

## Author Contributions

AS conducted all experiments and data analyses, co-designed all experimental procedures and co-wrote the manuscript. BA co-designed all experimental procedures and co-wrote the manuscript.

## Conflict of Interest Statement

The authors declare that the research was conducted in the absence of any commercial or financial relationships that could be construed as a potential conflict of interest.

## Footnotes

^1^dstolz.github.io/epsych/
